# Supplementary material for: Bisphenol BPAF and BPC are agonists for estrogen receptor ERα but antagonists for N-terminal domain-lacking ERα
Source: PLoS One. 2026 Jun 1;21(6):e0350499. doi: 10.1371/journal.pone.0350499 (PMC13225341; doi:10.1371/journal.pone.0350499)
Supplement: S1 Fig — (PDF) [file pone.0350499.s001.pdf]

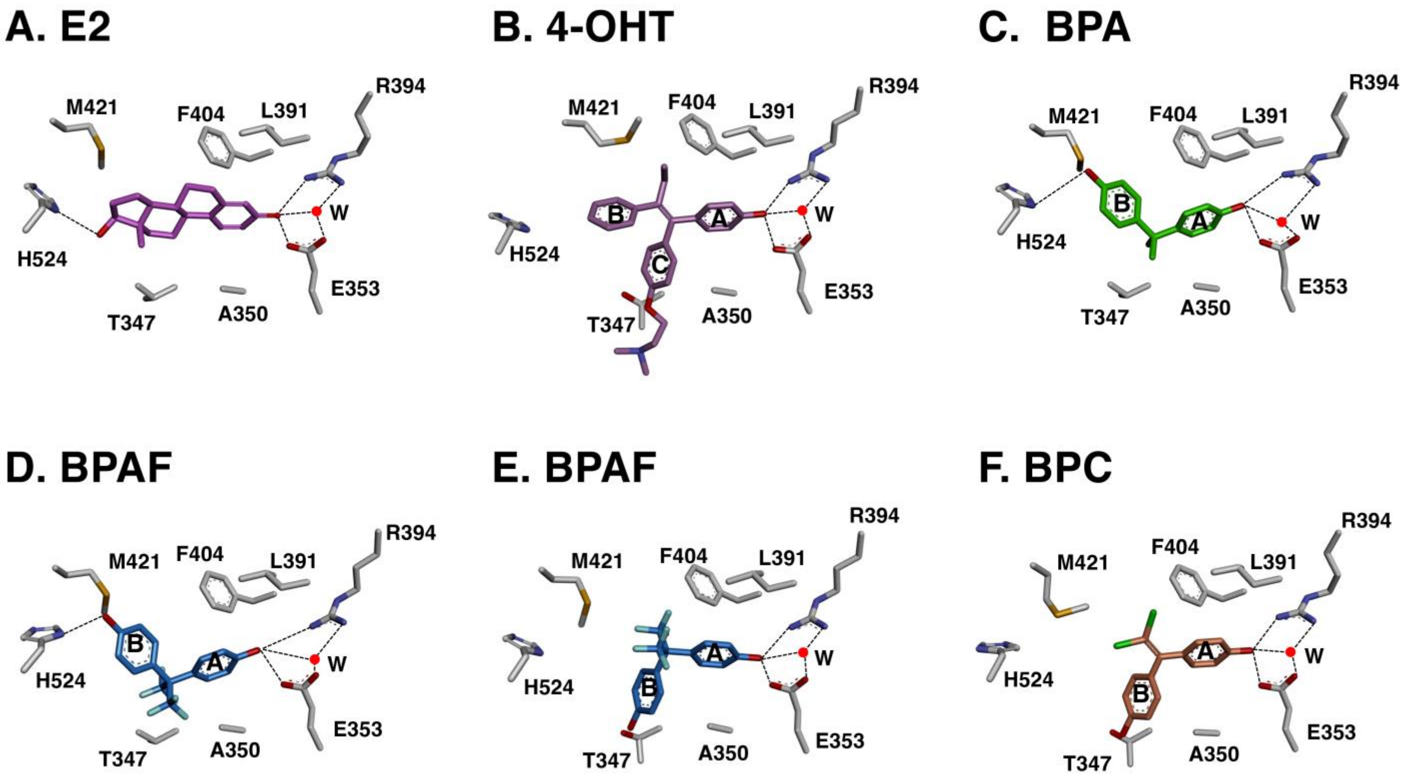

S1 Fig. ERα-LBD receptor-binding mode of a series of ligands. Y537S ERα-LBD is in complex with E2 (**A**), 4-OHT (**B**), BPA (**C**), BPAF (**D** and **E**), and wild-type ERα-LBD in complex with BPC (**F**). Structures were visualized and analyzed using Discovery Studio software from the PDB (ID codes: (**A**) 3UUD, (**B**) 7UJ8, (**C**) 3UU7, (**D** and **E**) 3UUA, and (**F**) 3UUC).
