# Supplementary material for: Bisphenol BPAF and BPC are agonists for estrogen receptor ERα but antagonists for N-terminal domain-lacking ERα
Source: PLoS One. 2026 Jun 1;21(6):e0350499. doi: 10.1371/journal.pone.0350499 (PMC13225341; doi:10.1371/journal.pone.0350499)
Supplement: S2 Fig — (PDF) [file pone.0350499.s002.pdf]

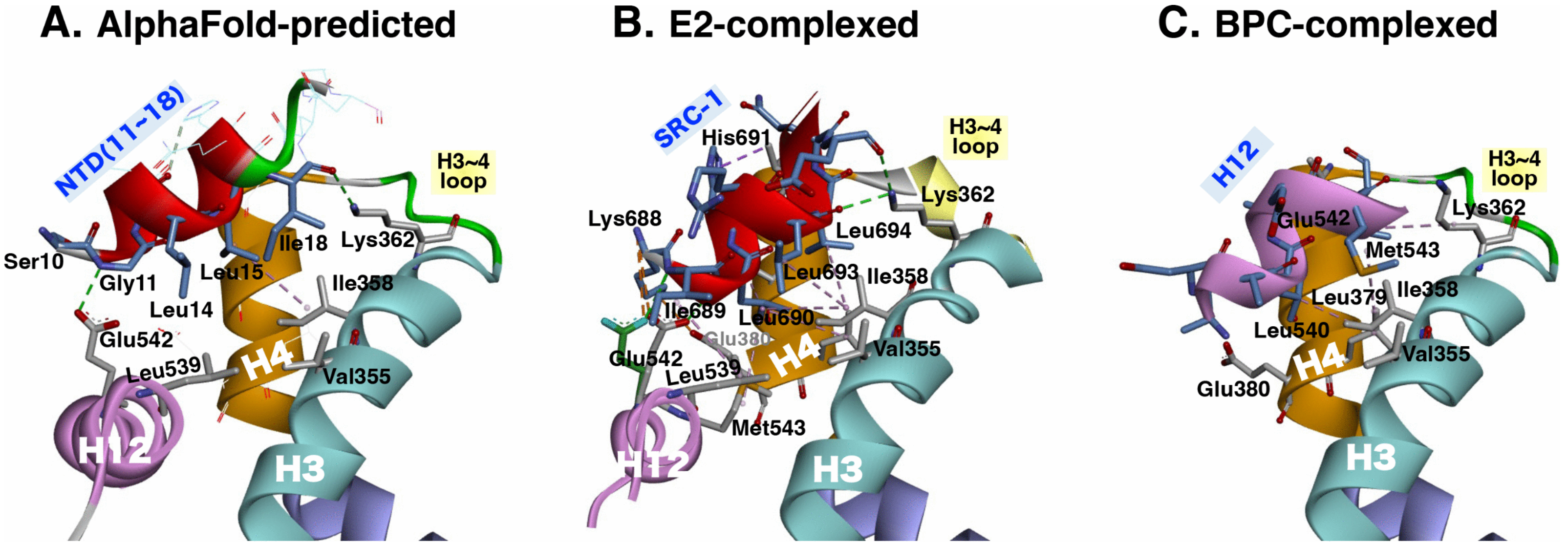

S2 Fig. Interaction networks of α-helix structures on the molecular surface of the C-terminal ERα-LBD(AF-2) domain. Analyzed α-helix structures: (**A**) AlphaFold-predicted NTD(11–18)-coupled LBD derived from full-length ERα with no ligand (P03372), (**B**) SRC-1 NR2 peptide (SRC-1)-coupled LBD in complex with E2 (from PDB 3UUD), and (**C**) H12 α-helix peptide in the antagonistic positioning of LBD in complex with BPC (from PDB 3UUC).
