# Supplementary material for: Bisphenol BPAF and BPC are agonists for estrogen receptor ERα but antagonists for N-terminal domain-lacking ERα
Source: PLoS One. 2026 Jun 1;21(6):e0350499. doi: 10.1371/journal.pone.0350499 (PMC13225341; doi:10.1371/journal.pone.0350499)
Supplement: S1 Table — (PDF) [file pone.0350499.s003.pdf]

S1 Table. Data for Schild plot analysis of the antagonist BPAF and BPC with desNTD(AF-1)-ERα.

Dose of administered antagonist [M]	Transcriptional inhibitory activity of antagonist BPAF and BPC against natural agonist E2[a]			
	BPAF		BPC	
	EC_{50} (nM) of E2	Log (DR – 1)[b]	EC_{50} (nM) of E2	Log (DR – 1)[b]
0	0.66 ± 0.07	————	0.66 ± 0.07	————
1.0×10^{-8}	1.70 ± 0.20	0.012 ± 0.0021	1.51 ± 0.28	0.057 ± 0.011
$1.0 \times 10^{-7.5}$	2.43 ± 0.17	0.115 ± 0.022	2.91 ± 0.18	0.313 ± 0.023
1.0×10^{-7}	3.57 ± 0.44	0.385 ± 0.025	4.00 ± 0.70	0.580 ± 0.078
$1.0 \times 10^{-6.5}$	6.89 ± 0.54	0.800 ± 0.14	11.2 ± 1.82	1.02 ± 0.20
1.0×10^{-6}	10.7 ± 1.26	1.01 ± 0.15	15.4 ± 1.74	1.25 ± 0.20
$1.0 \times 10^{-5.5}$	36.6 ± 4.79	1.37 ± 0.16	46.1 ± 2.66	1.74 ± 0.16
1.0×10^{-5}	41.7 ± 4.04	1.63 ± 0.19	93.1 ± 9.68	1.99 ± 0.13

[a]Data are presented as the mean ± SD estimated from at least three independent experiments (n≥3).

[b]The agonist dose ratio DR depends on the antagonist concentration [B] according to the relation DR = 1 + [B]/K_B, where K_B is the dissociation constant of the antagonist. The dose ratio DR is the ratio of the agonist concentration required for a half-maximal response with the antagonist present divided by the agonist required for half-maximal response without the antagonist ("control"). In other words, the ratio of the EC_{50} values of the inhibited and uninhibited curves. The equation [$\mathrm{Log}_{10}(DR - 1) = \mathrm{Log}_{10}[B] - \mathrm{Log}_{10} K_B$] is used to quantify the strength of the antagonist.
