# Supplementary material for: Bisphenol BPAF and BPC are agonists for estrogen receptor ERα but antagonists for N-terminal domain-lacking ERα
Source: PLoS One. 2026 Jun 1;21(6):e0350499. doi: 10.1371/journal.pone.0350499 (PMC13225341; doi:10.1371/journal.pone.0350499)
Supplement: S2 Table — (PDF) [file pone.0350499.s004.pdf]

S2 Table. Data for Schild plot analysis of 4-hydroxytamoxifen (4-OHT) with full-length ERα and desNTD(AF-1)-ERα.

Dose of administered 4-OHT [M]	Transcriptional inhibitory activity of antagonist 4-OHT against natural agonist E2			
	Full-length ERα		desNTD(AF-1)-ERα	
	EC_{50} (nM) of E2	Log (DR – 1)[b]	EC_{50} (nM) of E2	Log (DR – 1)[b]
0	0.63 ± 0.02	————	0.66 ± 0.07	————
1.0×10^{-8}	2.55 ± 0.45	0.275 ± 0.06	1.51 ± 0.12	0.163 ± 0.13
$1.0 \times 10^{-7.5}$	5.18 ± 1.17	0.753 ± 0.16	3.57 ± 0.48	0.700 ± 0.18
1.0×10^{-7}	11.5 ± 1.37	1.15 ± 0.23	10.9 ± 1.57	1.21 ± 0.22
$1.0 \times 10^{-6.5}$	37.4 ± 6.50	1.80 ± 0.16	42.3 ± 3.23	1.86 ± 0.27
1.0×10^{-6}	211 ± 25.4	2.41 ± 0.26	387 ± 41.2	2.72 ± 0.19
$1.0 \times 10^{-5.5}$	1510 ± 98.3	3.25 ± 0.30	1170 ± 156	3.29 ± 0.21
1.0×10^{-5}	4490 ± 391	3.74 ± 0.25	5140 ± 364	3.90 ± 0.21

[a]Data are presented as the mean ± SD estimated from at least three independent experiments (n≥3).

[b]The agonist dose ratio DR depends on the antagonist concentration [B] according to the relation DR = 1 + [B]/K_B, where K_B is the dissociation constant of the antagonist. The dose ratio DR is the ratio of the agonist concentration required for a half-maximal response with the antagonist present divided by the agonist required for half-maximal response without the antagonist ("control"). In other words, the ratio of the EC_{50} values of the inhibited and uninhibited curves. The equation [$\mathrm{Log}_{10}(DR - 1) = \mathrm{Log}_{10}[B] - \mathrm{Log}_{10} K_B$] is used to quantify the strength of the antagonist.
