# Supplementary material for: Bisphenol BPAF and BPC are agonists for estrogen receptor ERα but antagonists for N-terminal domain-lacking ERα
Source: PLoS One. 2026 Jun 1;21(6):e0350499. doi: 10.1371/journal.pone.0350499 (PMC13225341; doi:10.1371/journal.pone.0350499)
Supplement: S3 Table — (PDF) [file pone.0350499.s005.pdf]

S3 Table. Data for Schild plot analysis of the antagonist ICI 182,780 (ICI) with full-length ERα and desNTD(AF-1)-ERα.

Dose of administered ICI [M]	Transcriptional inhibitory activity of antagonist ICI against natural agonist E2[a]			
	Full-length ERα		desNTD(AF-1)-ERα	
	EC_{50} (nM) of E2	Log (DR – 1)[b]	EC_{50} (nM) of E2	Log (DR – 1)[b]
0	0.63 ± 0.02	————	0.66 ± 0.07	————
1.0×10^{-8}	1.34 ± 0.21	0.181 ± 0.017	1.60 ± 0.09	0.115 ± 0.019
$1.0 \times 10^{-7.5}$	3.89 ± 0.67	0.543 ± 0.087	3.80 ± 0.36	0.638 ± 0.075
1.0×10^{-7}	8.15 ± 1.78	1.04 ± 0.18	10.5 ± 1.38	1.14 ± 0.24
$1.0 \times 10^{-6.5}$	20.1 ± 3.50	1.46 ± 0.16	55.7 ± 4.09	1.89 ± 0.14
1.0×10^{-6}	70.5 ± 6.80	1.98 ± 0.30	332 ± 46.2	2.61 ± 0.33
$1.0 \times 10^{-5.5}$	510 ± 61.1	2.86 ± 0.24	956 ± 63.2	3.14 ± 0.24
1.0×10^{-5}	5300 ± 342	3.48 ± 0.55	5200 ± 406	3.87 ± 0.27

[a]Data are presented as the mean ± SD estimated from at least three independent experiments (n≥3).

[b]The agonist dose ratio DR depends on the antagonist concentration [B] according to the relation $DR = 1 + [B]/K_B$, where K_B is the dissociation constant of the antagonist. The dose ratio DR is the ratio of the agonist concentration required for a half-maximal response with the antagonist present divided by the agonist required for half-maximal response without the antagonist ("control"). In other words, the ratio of the EC_{50} values of the inhibited and uninhibited curves. The equation [$\mathrm{Log}_{10}(DR - 1) = \mathrm{Log}_{10}[B] - \mathrm{Log}_{10} K_B$] is used to quantify the strength of the antagonist.
