# Supplementary material for: Bisphenol BPAF and BPC are agonists for estrogen receptor ERα but antagonists for N-terminal domain-lacking ERα
Source: PLoS One. 2026 Jun 1;21(6):e0350499. doi: 10.1371/journal.pone.0350499 (PMC13225341; doi:10.1371/journal.pone.0350499)
Supplement: Attachment — Submitted filename: Response to Reviewers.pdf [file pone.0350499.s006.pdf]

Responses to Reviewers

April 23, 2026

Journal: PLOS ONE

Manuscript ID: PONE-D-26-08447

Title:
Bisphenol BPAF and BPC are agonists for estrogen receptor ERα but antagonists for N-terminal domain-lacking ERα

Authors:
Xiaohui Liu, Miki Shimohigashi, and Yasuyuki Shimohigashi

Dear Dr. Alavi and Reviewers,

Thank you very much for giving us the opportunity to revise our manuscript submitted to the journal of PLOS ONE. We appreciate the constructive feedback provided by the editors and reviewers. We present here the reply to all the comments and suggestions to our paper, and we believe that the manuscript has been improved fundamentally and is now acceptable to PLOS ONE.

Index

Titles	Page
I. Responses to the academic editor	2
II. Responses to Journal requirements	4
III. Responses to additional editor comments	8
IV. Responses to Reviewers	11

I. Responses to the academic editor

Blue: Comments and suggestions from the academic editor.

Black: Our point-by-point response.

❖ Please include the following items when submitting your revised manuscript:

- A letter that responds to each point raised by the academic editor and reviewer(s). You should upload this letter as a separate file labeled 'Response to Reviewers'.

Answer:

We are now providing a detailed, point-by-point response to all comments and suggestions raised by the Editors and Reviewers and also by the Journal requirements. This document has been uploaded as a separate file labeled '**Response to Reviewers**'.

- A marked-up copy of your manuscript that highlights changes made to the original version. You should upload this as a separate file labeled 'Revised Manuscript with Track Changes'.

Answer:

A marked-up version of the manuscript has been prepared and provided to the submission system. In this file, all changes to the original version were highlighted in yellow, with corrected words, sentences, or numbers in red. The file is labeled '**Revised Manuscript with Track Changes**'.

- An unmarked version of your revised paper without tracked changes. You should upload this as a separate file labeled 'Manuscript'.

Answer:

We did upload an unmarked version of our revised paper without tracked changes. The file was prepared in a separate file labeled '**Manuscript**'.

❖ If you would like to make changes to your financial disclosure, please include your updated statement in your cover letter. Guidelines for resubmitting your figure files are available below the reviewer comments at the end of this letter.

Answer:

There are no changes to our financial disclosure.

Regarding the figures, we have utilized the PACE tool and NAAS tool to ensure all files meet the journal’s technical requirements and guidelines.

❖ If applicable, we recommend that you deposit your laboratory protocols in protocols.io to enhance the reproducibility of your results. Protocols.io assigns your protocol its own identifier (DOI) so that it can be cited independently in the future. For instructions see: https://journals.plos.org/plosone/s/submission-guidelines#loc-laboratory-protocols.

Additionally, PLOS ONE offers an option for publishing peer-reviewed Lab Protocol articles, which describe protocols hosted on protocols.io. Read more information on sharing protocols at https://plos.org/protocols?utmmedium=editorial-email&utm_source=authorletters&utm_campaign=protocols.

Answer:

Thank you very much for the valuable information about the Lab Protocol. We have ensured that our 'Materials and Methods' section is described very in detail to certify reproducibility, and therefore we have decided not to deposit a separate protocol at this time. Meanwhile, we do not have any specialized deposits of Lab Protocol articles in the laboratory, and thus we do not intend to ask you for a proposed option.

❖ As the corresponding author, your ORCID iD is verified in the submission system and will appear in the published article. PLOS supports the use of ORCID, and we encourage all coauthors to register for an ORCID iD and use it as well. Please encourage your coauthors to verify their ORCID iD within the submission system before final acceptance, as unverified ORCID iDs will not appear in the published article. Only the individual author can complete the verification step; PLOS staff cannot verify ORCID iDs on behalf of authors.

Answer:

We have noted the importance of ORCID iDs for the visibility of the published work. Among corresponding authors, the first author Xiaohui Liu has already verified her ORCID iD as shown in the submission system. On the other hand, other co-authors do not have ORCID iDs at this point and do not plan to register for them in this submission. We understand that only verified IDs will be included in the final publication.

II. Responses to Journal requirements

Blue: Journal requirements.

Black: Our point-by-point response.

When submitting your revision, we need you to address these additional requirements.

1. Please ensure that your manuscript meets PLOS ONE's style requirements, including those for file naming. The PLOS ONE style templates can be found at https://journals.plos.org/plosone/s/file?id=wjVg/PLOSOne_formatting_sample_main_body.pdf and https://journals.plos.org/plosone/s/file?id=ba62/PLOSOne_formatting_sample_title_authors_affiliations.pdf.

Answer:

We have carefully reviewed the PLOS ONE style templates and ensured that the revised manuscript, including the title page, affiliations, main body, and file naming, strictly adheres to the journal's requirements.

2. Please note that PLOS One has specific guidelines on code sharing for submissions in which author-generated code underpins the findings in the manuscript. In these cases, we expect all author-generated code to be made available without restrictions upon publication of the work. Please review our guidelines at https://journals.plos.org/plosone/s/materials-and-software-sharing#loc-sharing-code and ensure that your code is shared in a way that follows best practice and facilitates reproducibility and reuse.

Answer:

We have reviewed the code sharing guidelines. In this study, the authors did not develop any custom code to support the results. All data analysis was performed using standard commercial software GraphPad Prism, and therefore, there is no custom code to be deposited.

3. Thank you for stating the following financial disclosure:

   "This work was supported by the Japan Society for the Promotion of Science (JSPS) KAKENHI grant nos. 25K15462, 22K12395, 19K12340, 15K00557, and 25740024 (to X.L.) and 22221005 and 15H01741 (to Y.S.). This work was also supported in part by a Health and Labour Sciences Research Grant for Research on the Risk of Chemical Substances from the Ministry of Health, Labor, and Welfare of Japan, under contract/grant no. H20-Chemistry-General-003 (to Y.S.)."

   Please state what role the funders took in the study. If the funders had no role, please state: "The funders had no role in study design, data collection and analysis, decision to publish, or preparation of the manuscript."

   If this statement is not correct you must amend it as needed.

   Please include this amended Role of Funder statement in your cover letter; we will change the online submission form on your behalf.

Answer:

Thank you very much for your kind advice about the amendment for Role of Funder statement. According to your indication, we newly added the statement "**The funders had no role in study design, data collection and analysis, decision to publish, or preparation of the manuscript**" in our cover letter to the Academic Editor.

4. Thank you for stating the following financial disclosure:

   "This work was supported by the Japan Society for the Promotion of Science (JSPS) KAKENHI grant nos. 25K15462, 22K12395, 19K12340, 15K00557, and 25740024 (to X.L.) and 22221005 and 15H01741 (to Y.S.). This work was also supported in part by a Health and Labour Sciences Research Grant for Research on the Risk of Chemical Substances from the Ministry of Health, Labor, and Welfare of Japan, under contract/grant no. H20-Chemistry-General-003 (to Y.S.)."

   We note that you have provided funding information that is not currently declared in your Funding Statement. However, funding information should not appear in the Acknowledgments section or other areas of your manuscript. We will only publish funding information present in the Funding Statement section of the online submission form.

   Please remove any funding-related text from the manuscript and let us know how you would like to update your Funding Statement. Currently, your Funding Statement reads as follows:

   "This work was supported by the Japan Society for the Promotion of Science (JSPS) KAKENHI grant nos. 25K15462, 22K12395, 19K12340, 15K00557, and 25740024 (to X.L.) and 22221005 and 15H01741 (to Y.S.). This work was also supported in part by a Health and Labour Sciences Research Grant for Research on the Risk of Chemical Substances from the Ministry of Health, Labor, and Welfare of Japan, under contract/grant no. H20-Chemistry-General-003 (to Y.S.)."

   Please include your amended statements within your cover letter; we will change the online submission form on your behalf.

Answer:

Thank you for this clarification. We have removed the **Acknowledgments** section from the manuscript, because our **Acknowledgments** contained only the funding-related information and grant numbers which should appear in the formal Funding Statement. Regarding the Funding Statement itself, the information currently recorded is correct, and no further changes to the grant numbers or recipients are needed. We have included the final, consolidated Funding Statement (including the "Role of Funders" sentence) in our **Cover Letter**.

5. We note that your Data Availability Statement is currently as follows: "All relevant data are within the manuscript and its Supporting Information files"

   Please confirm at this time whether or not your submission contains all raw data required to replicate the results of your study. Authors must share the "minimal data set" for their submission. PLOS defines the minimal data set to consist of the data required to replicate all study findings reported in the article, as well as related metadata and methods (https://journals.plos.org/plosone/s/data-availability#loc-minimal-data-set-definition).

For example, authors should submit the following data:
- The values behind the means, standard deviations and other measures reported;
- The values used to build graphs;
- The points extracted from images for analysis.

Authors do not need to submit their entire data set if only a portion of the data was used in the reported study.

If your submission does not contain these data, please either upload them as Supporting Information files or deposit them to a stable, public repository and provide us with the relevant URLs, DOIs, or accession numbers. For a list of recommended repositories, please see https://journals.plos.org/plosone/s/recommended-repositories.

If there are ethical or legal restrictions on sharing a de-identified data set, please explain them in detail (e.g., data contain potentially sensitive information, data are owned by a third-party organization, etc.) and who has imposed them (e.g., an ethics committee). Please also provide contact information for a data access committee, ethics committee, or other institutional body to which data requests may be sent. If data are owned by a third party, please indicate how others may request data access.

Answer:

According to your indication, we described, "All data are in the manuscript and/or supporting information files." in the statement. We did not submit the entire data set, for example, to build the graphs shown Fig 2 and 3. This is because only certain portions of the definite solid data were used for the analyses. The minimum experimental analytical data are shown in figures and tables.

When necessary, we included the supporting data set necessary to build graphs. For instance, the Schild plot analyses shown in Fig 4b–6b were created from the EC_{50} data obtained from the dose-response curves shown in the same Figures; i.e., Figs 4a–6a. These EC_{50} data were shown in the information files of S1–S3 Tables, respectively. We believe that these are satisfactory enough to fulfill your above requirements.

We had no data set that are ethical or legal restrictions on sharing.

6. We notice that your supplementary figures are uploaded with the file type 'Figure'. Please amend the file type to 'Supporting Information'. Please ensure that each Supporting Information file has a legend listed in the manuscript after the references list.

Answer:

In the submission of the revised manuscript, we are going to gather all the supplementary figures and supplementary tables up in one file and submit it to '**Supporting Information**' in the submission system. Meanwhile, each Supporting Information captions are listed at the end of the manuscript, immediately following the References list, as required in the PLOS Manuscript Body Formatting Guidelines.

7. If the reviewer comments include a recommendation to cite specific previously published works, please review and evaluate these publications to determine whether they are relevant and should be cited. There is no requirement to cite these works unless the editor has indicated otherwise.

Answer:

We have noted the journal's policy regarding citation recommendations. Although the reviewers did not suggest any specific citations to be added, we added some explanations by citing our previous publications, some of which were already listed in the **References** and some others were not, especially to reply to the comment (III) by Reviewer #2.

III. Responses to additional editor comments

Blue: Additional editor comments.

Black: Our answer to the academic editor.

Red: Sentences added newly in the old Manuscript text.

- Please follow the PLOS ONE guidelines to organize the MS.

Answer:

Yes, we have carefully reviewed and followed the PLOS ONE manuscript organization guidelines and the PLOS Manuscript Body Formatting Guidelines. The revised manuscript has been restructured to ensure that all sections (Title Page, Abstract, Introduction, Materials and Methods, Results, Discussion, Conclusions, and References) appear in the correct order. We have also ensured that Figure Legends are placed immediately after the paragraph in which they are first cited, and that the Supporting Information captions are listed at the end of the manuscript, as required.

- Please clarify why $P < 0.001$ was considered for a significant difference; usually, $P < 0.05$ is taken into account.

Answer:

Simply, $P < 0.001$ was indicated by the software used. Our dose-response data showed an extremely high statistical significance or confidence, and thus simply in order to show this, we used $P < 0.001$ as indicated.

- Did you check the normal distribution and homogeneity of variance for *t*-Test?

Answer:

Yes, using the software GraphPad Prism 10, we verified the normality and homogeneity of variance. These procedures have been described in the **Materials and Methods** section. We have revised the description of ***Statistical analysis*** as follows.

page 8, line 242–245 → page 9, line 257–262 of Revised Manuscript with Track Changes version

Statistical analysis

All data are presented as mean ± SD for the indicated number of independent experiments. Statistical significance was determined using a two-tailed Student's t-test, with $p < 0.001$ considered significant.

Statistical analysis

All assays were performed at least three times ($n \geq 3$) in each independent experiment, with each sample concentration in duplicate. Data analyzed by the software GraphPad Prism 10 are presented as the mean ± standard deviation (SD) for the indicated number of independent experiments. Statistical significance was determined using a two-tailed Student's *t*-test with $p < 0.001$ considered significant.

- Was not it possible to show the significant differences on Figures or in Tables?

Answer:

At each data point in the Figures, the deviation estimated are shown with bars. In the Tables, the standard deviation (± SD) is shown alongside the analytical values [EC_{50}/IC_{50}/etc.]. It has been confirmed that these represent the significance levels at which a 'statistically significant difference' is deemed to exist.

- Please add number of experiments performed for each assay, both in the method, in the legend of figures, and in the title of Tables.

Answer:

Thank you for this valuable comment. According to your suggestions, we have now clearly indicated the number of independent experiments (n) in all relevant sections. Each assay was performed at least three times independently ($n \geq 3$). This information has been added to the Statistical analysis section of **Materials and Methods**, the Figure Legends (Figs 2-6), and the notes of Tables (Tables 1-3, and S1–S3 Tables).

- For funding: Please state what role the funders took in the study. If the funders had no role, please state: ""The funders had no role in study design, data collection and analysis, decision to publish, or preparation of the manuscript.""

Answer:

The fact is that the funders did not play any roles in the study design, data collection and analysis, decision to publish, or preparation of the manuscript. According to the comment from the academic editor, in addition to the Funding section in the online submission form, the following statement was included in our new **Cover Letter** for submitting the revised manuscript: "**The funders had no role in study design, data collection and analysis, decision to publish, or preparation of the manuscript**."

- Please clarify "Competing Interests Statement"

Answer:

We have confirmed that there are no competing interests to declare, and we have declared that no competing interests exist. This statement has been included also in our **Cover Letter**: "**The authors have declared that no conflicts of interest exist**."

- Please provide Ethical statement.

Answer:

All experiments in this study were conducted *in vitro* using HeLa cell lines. No human participants, human data, or animal subjects were involved in this research. Therefore, specific ethical approval from an institutional review board was not required for this study.

IV. Responses to Reviewers

Blue: Reviewer's comments.

Black: Our answer to the reviewer's comments.

Red: Sentences added newly in the old Manuscript text.

Reviewer's Responses to Questions
Comments to the Author

1. Is the manuscript technically sound, and do the data support the conclusions?

The manuscript must describe a technically sound piece of scientific research with data that supports the conclusions. Experiments must have been conducted rigorously, with appropriate controls, replication, and sample sizes. The conclusions must be drawn appropriately based on the data presented.

Reviewer #1: Yes
Reviewer #2: Yes

Answer:

We greatly appreciate your appropriate response "Yes," an affirmative reply to the questions about evaluation of our study.

2. Has the statistical analysis been performed appropriately and rigorously?

Reviewer #1: Yes
Reviewer #2: Yes

Answer:

We thank the reviewers for their right evaluation of our statistical methodology.

3. Have the authors made all data underlying the findings in their manuscript fully available?

The PLOS Data policy requires authors to make all data underlying the findings described in their manuscript fully available without restriction, with rare exception (please refer to the Data Availability Statement in the manuscript PDF file). The data should be provided as part of the manuscript or its supporting information, or deposited to a public repository. For example, in addition to summary statistics, the data points behind means, medians and variance measures should be available. If there are restrictions on publicly sharing data—e.g. participant privacy or use of data from a third party—those must be specified.

Reviewer #1: No
Reviewer #2: Yes

Answer:

We acknowledge the concern raised by Reviewer #1 regarding data availability. According to the comments from the academic editor, and especially in accordance with PLOS ONE's Data Policy, we have explained above about our data availability, in which we had provided the "minimal data set." Meanwhile, we had provided the full data set, for example, to build the graphs of Schild plot analyses. These data are given in the S1–S3 Tables, and these are required to replicate satisfactorily our findings. We have ensured that all underlying data are now fully available without restriction.

---

4. Is the manuscript presented in an intelligible fashion and written in standard English?

PLOS ONE does not copyedit accepted manuscripts, so the language in submitted articles must be clear, correct, and unambiguous. Any typographical or grammatical errors should be corrected at revision, so please note any specific errors here.

Reviewer #1: No
Reviewer #2: Yes

Answer:

While the original manuscript had undergone professional English proofreading prior to the initial submission, we took the comments from Reviewer #1 seriously. In response to this indication, we thoroughly revised the entire text to improve clarity and flow, and several sections to ensure there were no ambiguities. We are now confident that the revised manuscript fully meets the standard English requirements for publication in PLOS ONE.

5. Review Comments to the Author

Please use the space provided to explain your answers to the questions above. You may also include additional comments for the author, including concerns about dual publication, research ethics, or publication ethics. (Please upload your review as an attachment if it exceeds 20,000 characters)

Blue: Reviewer's comments.

Black: Our answer to the reviewer's comments.

Red: Sentences added newly in the old Manuscript text.

Reviewer #1: General comment:

1. All the figures, including those as supplementary ones, have to be presented with a better resolution.

Answer:

We regret to know the apparently poor resolution of the figures in our initial submission. We have now recreated all figures and supplementary figures at a higher resolution in accordance with PLOS ONE’s figure guidelines. All final figures have been verified using the PACE and NASS tool provided by PLOS to ensure they meet the required technical standards for publication.

Reviewer #1: Minor comments.

1. Line 110, Begin the sentence with: Indeed

Answer:

Yes, we agree with the suggestion to improve the flow of the paragraph. We have revised the sentence starting at Line 110 by adding "Indeed," at the beginning.

Page 4, line 110 → page 4, line 110 of Revised Manuscript with Track Changes version

110 transcriptional activity. This NTD(AF-1)-lacking ERα retains approximately 65% of wild-

↓

110 transcriptional activity. Indeed, this NTD(AF-1)-lacking ERα retains approximately 65%

2. Line 137, Remove the last dot.

Answer:

Yes, we removed the unnecessary dot at the end of the sentence on Line 137 as suggested.

page 5, line 137 → page 5, line 147 of Revised Manuscript with Track Changes version

137 ERα and desNTD(AF-1)-ERα..

↓

147 in full-length ERα and desNTD(AF-1)-ERα.

3. For clarity, add for Figure 1C, add below the structures the corresponding E2, 4-OH, ICI, BPA, BPAF, BPC.

Answer:

Yes, we agree to add the labels of compound name for the structures shown in Fig 1C. This improves significantly the clarity of the figure. As suggested, we have added the corresponding compound names (E2, 4-OHT, ICI, BPA, BPAF, and BPC) directly to the subtitles (**a.**, **b.**, **c.**, **d.**, **e.**, and **f.**, respectively) at above their respective chemical structures in the revised Fig 1C. We decided to place the names together with the subtitles, but not below the structures, simply because of improved readability and uniformity.

4. Line 250 to 252, simplify the sentence. Remove the semicolon in line 251 and write again the sentence.

Answer:

Yes, as suggested, we simplified the sentence by removing the semicolon and rephrasing it for better clarity and flow.

page 8–9, line 250–253 → page 9, line 267–270 of Revised Manuscript with Track Changes version

250 Wild-type full-length ERα and N-terminal NTD(AF-1)-lacking desNTD(AF-1)-ERα share
251 the same ligand-binding domain LBD(AF-2); therefore, all ligands would be expected to
252 bind to the ligand-binding pocket (LBP) with the same binding affinity for both full-length
253 ERα and desNTD(AF-1)-ERα.

↓

267 Because wild-type full-length ERα and N-terminal NTD(AF-1)-lacking
268 desNTD(AF-1)-ERα share the same ligand-binding domain LBD(AF-2), all ligands would
269 be expected to bind to the ligand-binding pocket (LBP) with the same binding affinity for
270 both full-length ERα and desNTD(AF-1)-ERα.

5. Line 331, “eventually reaching baseline”. Remove this part and be more precise.

Answer:

Yes, as suggested, we removed the phrase “eventually reaching baseline” and revised the sentence to provide a more precise description of the results.

page 11, line 329–332 → page 13, 357–361 of Revised Manuscript with Track Changes version

4-OHT was also a strong antagonist for desNTD(AF-1)-ERα. The dose–response curve depicting the activity of 10 nM E2 showed a clear decrease depending on the doses of 4-OHT, eventually reaching baseline (fold-activation = 1) with a 4-OHT concentration of 1.0 ×10^{-5} M (Fig. 3Ab). 4-OHT was a fully pure antagonist, with an IC_{50} value of 9.95 nM (Table 3).

↓

4-OHT was also a strong antagonist for desNTD(AF-1)-ERα. The dose–response curve depicting the activity of 10 nM E2 showed a clear decrease depending on the doses of 4-OHT, with transcriptional activity being inhibited entirely (the curve reached the basal level, namely, fold-activation = 1) with a 4-OHT concentration of 1.0 ×10^{-5} M (Fig. 3Ab). 4-OHT was a fully pure antagonist, with an IC_{50} value of 9.95 nM (Table 3).

6. Remove sentence from line 445 to 446.

Answer:

With this comment, we considered carefully the corresponding sentence, which describes “BPAF and BPC bind to the ERα-LBP present in LBD(AF-2). Several points are important to the ‘agonist-to-antagonist functional conversion.’” However, we believe that these sentences are certainly important to indicate and emphasize a key finding in this our study. Although BPAF and BPC bind to the same ligand-binding pocket (LBP) in both receptor types, their functional outcomes differ fundamentally, depending upon the presence or absence of the NTD.

In response to the comment, we decided to rewrite this section in a more concise manner to address concerns regarding the flow of the text. It is critically important to impress and interpret the phrase “agonist-to-antagonist functional conversion”, which was brought about or caused by the removal of the N-terminal 1–180 peptide fragment.

page 15, line 445–447 → page 18, 488–492 of Revised Manuscript with Track Changes version

BPAF and BPC bind to the ERα-LBP present in LBD(AF-2). Several points are important to the ‘agonist-to-antagonist functional conversion.’ The domain eliminated is the N-terminal 1–180 peptide fragment containing the activation function motif AF-1.

↓

Both BPAF and BPC bind to the same ligand-binding pocket (LBP) of

ERα-LBD(AF-2). These bisphenols were agonists for full-length ERα, but antagonists for desNTD(AF-1)-ERα. Here is a clear functional conversion, ‘agonist-to-antagonist’ upon the removal of the N-terminal 1–180 peptide fragment containing the activation function motif AF-1.

7. For Figure 7, indicate the helices number from 1 to 12.

Answer:

Yes, we appreciate the suggestion to label the helices number from 1 to 12. We have revised Fig 7 to include these numbering of the helices within the ERα-LBD.

Blue: Reviewer's comments.

Black: Our answer to the reviewer's comments.

Red: Sentences added newly in the old Manuscript text.

Reviewer #2:

(I)

- This manuscript describes and investigation of binding and functional properties of bis-phenol A (BPA), the bis-trifluoromethylated analog (BPAF) and a gem-dichloroethylene analog (BPC) with wild-type Estrogen Receptor alpha and the modified protein that lacks the N-terminal domain [NTD(AF-1)]. The major findings are that the modified protein retains a significant amount of transcriptional activity relative to the full-length protein, however, while the simple parent compound BPA is an agonist for both proteins, the halogenated small molecules behave as antagonists in the absence of the N-terminal domain. The paper is written clearly, the experiments are described in sufficient detail, and the references are appropriate.

Answer:

We greatly appreciate your very appropriate comprehension and interpretation of our paper with high evaluation. In particular, we thank very much for the comment and judgment that “The paper is written clearly, the experiments are described in sufficient detail, and the references are appropriate.”

(II)

- This concluding statement is unsatisfactory and needs further elaboration: “The driving force for this H12 translocation is likely due to the halogen atoms present in BPAF and BPC, and its effect is only fully observed upon NTD(AF-1) removal.”

Answer:

We greatly appreciate your very appropriate comment and suggestion.

The corresponding concluding statement is "The driving force for this H12 translocation is likely due to the halogen atoms present in BPAF and BPC, and its effect is only fully observed upon NTD(AF-1) removal." For further detailed elaboration, we revised this concluding statement in the **Conclusions** section as follows.

"The driving force for this H12 translocation from the agonist positioning to the antagonist positioning is likely due to the specific halogen bonding by the CF_3 groups in BPAF and the Cl atoms in BPC. These halogen bondings eventually appear to destruct the hydrophobic bondings between the H11 and H12. However, the effect of halogen bondings to release H12 to the antagonist positioning was shown only when NTD(AF-1) was removed, and therefore it is strongly suspected that the interaction between N-terminal NTD(AF-1) and C-terminal LBD(AF-2) works to block the effect of halogen bondings."

In the manuscript, the revision became as follows.
page 24, **Conclusions**, line 726–728→
page 28, 776–783 of Revised Manuscript with Track Changes version

The driving force for this H12 translocation is likely due to the halogen atoms present in BPAF and BPC, and its effect is only fully observed upon NTD(AF-1) removal.

The driving force for this H12 translocation from the agonist positioning to the antagonist positioning is likely due to the specific halogen bonding by the CF_3 groups in BPAF and the Cl atoms in BPC. These halogen bondings eventually appear to destruct the hydrophobic bondings between the H11 and H12. However, the effect of halogen bondings to release H12 to the antagonist positioning was shown only when NTD(AF-1) was removed, and therefore it is strongly suspected that the interaction between N-terminal NTD(AF-1) and C-terminal LBD(AF-2) works to block the effect of halogen bondings.

(III)

❖ The significance of these findings could be clarifies and strengthened for the reader by addressing several related questions. What is the broader context of these specific results of bisphenol-related environmental estrogens within the classical example of estrogen receptor alpha (ERα) as a ligand-dependent transcription factor and why should we care?

Answer:

Thank you very much for your comment "The significance of these findings could be clarifies and strengthened for the reader by addressing several related questions." We agree to describe the answer to the questions "What is the broader context of these specific results of bisphenol-related environmental estrogens within the classical example of estrogen receptor alpha (ERα) as a ligand-dependent transcription factor?" and "why we should care?"

The most important fundamental facts known to date are the followings.

(1) Bisphenol A (BPA) is a so-called endocrine-disrupting chemical, which had been suspected to interact with endocrine hormone receptor ERα.

(2) We discovered a novel nuclear receptor named 'estrogen-related receptor gamma (ERRγ)' as its high-affinity target, which is highly expressed in human brain and endocrine system. Our another finding that ERRγ is most abundantly expressed in the human placenta has highlighted the BPA's high potential as a health risk.

(3) So-called next-generation bisphenols were developed as a substitute of BPA during these several decades, and BPAF and BPC are such representative compounds. However, these bisphenols have been found to interact also with nuclear receptors such as ERα, ERβ, and ERRγ.

Although the essences of these facts were mostly described already in the section of **Introduction**, we decided to expand substantially the description in **Introduction**, providing a more comprehensive context. There are two major changes in **Introduction** as follows.

(I) One sentence was inserted,
page 4, lines 119–127 → page 4, 119–129 of Revised Manuscript with Track Changes version

We previously reported that potent endocrine-disrupting chemicals such as bisphenol AF (BPAF) and bisphenol C (BPC) (Fig. 1C), as well as other bisphenols, function as agonists for ERα but as antagonists for ERβ [16–19]. ERβ possesses an NTD(AF-1) domain that is 36 residues shorter than that of ERα-NTD(AF-1). Although the amino acid sequences of the NTD(AF-1) domains are dissimilar, the DBD and LBD(AF-2) domains of ERα and ERβ are highly similar. We hypothesized that the ERα-agonist/ERβ-antagonist activity of BPAF and BPC might be due to a difference in the N-terminal NTD(AF-1) domains. This hypothesis prompted us to examine the activity of a series of estrogens and xenoestrogens against desNTD(AF-1)-ERα.

↓

We previously reported that potent endocrine-disrupting chemicals such as bisphenol AF (BPAF) and bisphenol C (BPC) (Fig. 1C), as well as other bisphenols, function as agonists for ERα but as antagonists for ERβ [16–19]. ERβ possesses an NTD(AF-1) domain that is 36 residues shorter than that of ERα-NTD(AF-1). BPAF and BPC were found to bind to ERα and ERβ considerably strongly, and thus they were with a robust concern for their endocrine-disturbing action in the human body [20]. Although the amino acid sequences of the NTD(AF-1) domains are dissimilar, the DBD and LBD(AF-2) domains of ERα and ERβ are highly similar. We hypothesized that the ERα-agonist/ERβ-antagonist activity of BPAF and BPC might be due to a difference in the N-terminal NTD(AF-1) domains. This hypothesis prompted us to examine the activity of a series of estrogens and xenoestrogens against desNTD(AF-1)-ERα.

(II) Several sentences were inserted,
page 4, lines 128–137 → page 5, 130–147 of Revised Manuscript with Track Changes version

In the present study, we evaluated the intrinsic receptor characteristics of ERα and desNTD(AF-1)-ERα using a traditional agonist and antagonists for wild-type ERα: E2, 4-hydroxytamoxifen (4-OHT), and ICI 182,780 (ICI) (Fig. 1C). We also assayed bisphenol A (BPA), a chemical compound primarily used in the manufacture of various plastics. BPA is a xenoestrogen and endocrine disruptor [20] that mimics the effects of estrogen in the body. BPAF and BPC (Fig. 1C), members of the next-generation bisphenol family, were also tested against ERα and desNTD(AF-1)-ERα. We describe the receptor binding affinity, transcriptional activation activity, and antagonist activity of these estrogens and xenoestrogens, and discuss the molecular mechanisms of receptor responses in full-length ERα and desNTD(AF-1)-ERα.

In the present study, we evaluated the intrinsic receptor characteristics of ERα and desNTD(AF-1)-ERα using a traditional agonist and antagonists for wild-type ERα: E2, 4-hydroxytamoxifen (4-OHT), and ICI 182,780 (ICI) (Fig. 1C). We also assayed bisphenol A (BPA), a chemical compound primarily used in the manufacture of various plastics. BPA is a xenoestrogen and endocrine disruptor [21] that mimics the effects of estrogen in the body. We found that BPA binds strongly to human estrogen-related receptor gamma (ERRγ), but only weakly to ERα [22, 23]. We further demonstrated that ERRγ, one of 48 human nuclear receptors, is most abundantly expressed in the human placenta [24]. In these a few decades, serious concern is the leakage of BPA and its substitutes from chemical products. In particular, a strong apprehension has been raised about crucial endocrine-disrupting effects to human health, especially as the adverse impacts on the development of the reproductive systems and fetal brain. BPAF and BPC bind to ERRγ fairly strongly, and now ERα, ERβ, and ERRγ appear to be the major targets of bisphenols among human endocrine hormone receptors [17, 19, 25–27]. In the present study, BPA, BPAF and BPC were tested against ERα and desNTD(AF-1)-ERα. We describe the receptor binding affinity, transcriptional activation activity, and antagonist activity of these estrogens and xenoestrogens, and discuss the molecular mechanisms of receptor responses in full-length ERα and desNTD(AF-1)-ERα.

<u>Next three literatures were added newly in the Reference list, and therefore literature number following these increase by 3.</u>

22. Liu X, Matsushima A, Okada H, Shimohigashi Y. Distinction of the binding modes for human nuclear receptor ERRgamma between bisphenol A and 4-hydroxytamoxifen. J Biochem. 2010; 148(2):247–254. https://doi.org/10.1093/jb/mvq056. PMID: 20542892.

23. Okada H, Tokunaga T, Liu X, Takayanagi S, Matsushima A, Shimohigashi Y. Direct evidence revealing structural elements essential for the high binding ability of bisphenol A to human estrogen-related receptor-gamma. Environ Health Perspect. 2008; 116(1):32–38. https://doi.org/ 10.1289/ehp.10587. PMID: 18197296.

24. Takeda Y, Liu X, Sumiyoshi M, Matsushima A, Shimohigashi M, Shimohigashi Y. Placenta expressing the greatest quantity of bisphenol A receptor ERR gamma among the human

reproductive tissues: Predominant expression of type-1 ERR gamma isoform. J Biochem. 2009; 146(1):113–122. https://doi.org/10.1093/jb/mvp049. PMID: 19304792.

(IV)

- What are the major structural/steric differences between these compounds evaluated and how are the halogens involved, given their ability to modify properties such as acidity through induction, and the capacity of fluorinated alkanes to act as weak hydrogen bond acceptors? Are there known differences in the biological or toxicological activity profiles of the compounds?

Answer:

Thank you for providing such insightful comments on the compounds evaluated, BPAF and BPC. Asked questions are (1) What are the major structural/steric differences between these compounds ?, (2) how are the halogens involved, given their ability to modify properties such as acidity through induction, and the capacity of fluorinated alkanes to act as weak hydrogen bond acceptors?, and (3) Are there known differences in the biological or toxicological activity profiles of the compounds?

In the present study, we newly found that Bisphenol BPAF and BPC are agonists for estrogen receptor ERα but antagonists for N-terminal domain-lacking ERα. These our novel results revealed that the presence of the N-terminal NTD(AF-1) domain substantiates the agonist activity of these halogen-containing BPAF and BPC in wild-type full-length ERα and that ERα-NTD(AF-1) plays an essential role in determining the agonist/antagonist activity of BPAF and BPC for estrogen receptor ERα.

However, although the driving force for this H12 translocation from the agonist positioning to the antagonist positioning is likely due to the specific halogen bondings by the CF_3 groups in BPAF and the Cl atoms in BPC molecules, it is not clear what kind specific halogen bonding interactions are responsible for the destruction of the hydrophobic bondings between the H11 and H12. To place or translocate the H12 to the antagonist positioning, the destruction of the hydrophobic bondings between the H11 and H12 is absolutely necessary.

The motive power of halogen bonding is fully displayed for ERα only when NTD(AF-1) is removed. Therefore, further investigation is required to elucidate the complete picture of the interaction(s) between NTD(AF-1) and LBD(AF-2) in ERα. All these future necessities have described in the text in detail.

Under these circumstances, it is very difficult to answer to the above questions from the reviewer #2. This is especially because we have not clarified yet any specific details about halogen bondings between the BPAF or BPC molecule and ERα-ligand-binding site, and slso any specific structural requirements for the H12 translocation from the agonist positioning to the antagonist positioning.

We described in the text (lines 749–751 in Revised Manuscript with Track Changes version manuscript) “Our recent investigation to identify similar interactions between NTD(AF-1) and LBD(AF-2) has revealed at least three interactions, including one involving the NTD(11–18) α-helix peptide. These investigations are under way and will be reported in due course.”

We do promise to describe the answers to the reviewer’s questions, especially revealing the whole figures of structural/steric characteristics of BPAF and BPC and their specific halogen bondings.

Meanwhile, in order to make these points clear for improvement of the current paper, it is evident that we need a continuing investigation. Thus, we decided to add the several sentences about the future studies at the end of the revised manuscript at the **Conclusion** section (page 28–29). The sentences added are “In conclusion, further investigation is necessary to elucidate the complete picture of the actual formations of halogen bondings, and also of the actual molecular mechanism of the H12-translocation. We will expose the structural characteristics of BPAF and BPC in their halogen bondings with the ligand-binding site of ERα.”

In the manuscript, the revision became as follows.
page 24, **Conclusions**, lines 747–754 → page 28–29, 784–791 of Revised Manuscript with Track Changes version

Elucidating the role of NTD(AF-1) in regulating H12 translocation is of significant interest. The present results may provide insight into the NTD(AF-1)-specific molecular mechanism underlying the antagonistic effects of BPAF and BPC on desNTD(AF-1)-ERα, and conversely, the agonistic effects of BPAF and BPC on full-length ERα.

⬇

In conclusion, further investigation is necessary to elucidate the complete picture of the actual formations of halogen bondings, and also of the actual molecular mechanism of the H12-translocation. We will expose the structural characteristics of BPAF and BPC in their halogen bondings with the ligand-binding site of ERα. In particular, elucidating the role of NTD(AF-1) in regulating H12 translocation is of significant interest. The present results may provide insight into the NTD(AF-1)-specific molecular mechanism underlying the antagonistic effects of BPAF and BPC on desNTD(AF-1)-ERα, and conversely, the agonistic effects of BPAF and BPC on full-length ERα.

For reference to Reviewer #2, we would like to note the followings.

Regarding BPC, our paper (lit #19: PLOS ONE | https://doi.org/10.1371/journal.pone.0246583, 2021) provides a very detailed explanations and illustrations, particularly concerning the molecular mechanisms underlying the formation of halogen bondings and the characteristics of their electronic

chemical structures. Please refer the **Discussion** section of this our PLOS paper, with the following Figures.

Fig 7. Schematic diagram of halogen bonding. Halogen bonds exist between the halogen atom X (F, Cl, Br, or I) and the electron-rich δ– and/or electron-poor δ+ sites in the receptor protein. The electrons of atom X in the ligand molecule are maldistributed to the electron-rich δ– and electron-poor δ+ portions.

Fig 8. Interactive chemical structure models of BPC. Chemical constitution formulas are shown together with the electronic formula of chlorine and oxygen atoms (**A**), while the canonical forms or resonance structures are expressed for the vinylidene chloride moiety of BPC (**B**).

In this PLOS ONE paper, we introduced the nature of halogen bondings. Halogen bonding is a type of electrostatic interaction, which is the interaction between positive and negative charges. Halogen bonding depends on the dispersion force of halogen atoms, and its the ascending strength order is F < Cl < Br < I. Although the activity ascending order of BPAF < BPC should be explained fundamentally by the halogen bonding with the receptors as a fundamental driving force, the activity strength of BPAF and BPC must be explained by their some other structural characteristics.

BPAF is a substitute of BPA, but it has also been assume to be an endocrine disruptor. BPAF contains two trifluoromethyl (CF_3) groups, each consisting of three strongly polar C–F bonds which form a substructure with very high local polarity. Each C–F bond carries a partial positive charge on the C atom side and a partial negative charge on the F atom side. As a result, the central C atom is in the **tetrahedral** (sp^3-hybridized) structure, and considerably strongly charged positively, although it is highly hindered sterically. Thus, CF_3 group interacts with surrounding polar groups by "electrostatic attraction" and "repulsion."

(V)

❖ The fact that there are numerous clinically significant mutations in the ER's ligand-binding domain that are relevant for cancer raises the potential here to include discussion of known mutations and structural variants in or involving the N-terminal domain (NTD) that contribute to pathological conditions.

Answer:

Thank you very much for your very valuable comments about the mutations and structural variants in ERα. If there are numerous clinically significant mutations in the ER's ligand-binding domain and if those mutations are relevant for cancer, it is definitely important to include such mutations in the discussion. Unfortunately, however, specific mutations within the ligand-binding domain that directly alter the receptor responses of these halogenated BPAF and BPC bisphenols

have not been reported in the literature to date. Therefore, it is difficult to discuss such specific mutations in detail at this stage.

On the other hand, naturally occurring ERα variants lacking specific domains are known in various pathological conditions, including breast cancer cells. Those include variants such as NTD-deficient isoforms and LBD-deficient isoforms. These structural variants are likely to alter the pharmacological responses to environmental estrogens. We are currently conducting investigations focusing on the interactions between these naturally occurring variants and halogenated bisphenols. We are expecting to report these findings in a quite near future.

(VI)

❖ One other minor comment is that in Table 1 the column titled relative "potency" should be changed to "affinity".

Answer:

Yes, "potency" should be "affinity." We appreciate this correction regarding pharmacological terminology. We have updated the column title in Table 1 from "relative potency" to "relative affinity" to accurately reflect the binding data presented.

---

6. PLOS authors have the option to publish the peer review history of their article (what does this mean?). If published, this will include your full peer review and any attached files.

If you choose "no", your identity will remain anonymous but your review may still be made public.

Do you want your identity to be public for this peer review? For information about this choice, including consent withdrawal, please see our Privacy Policy.

Reviewer #1: No
Reviewer #2: No

Answer:

We have no comment on this matter.

(End of document end of a sentence)
